# Special Issue on “Sphingolipids: From Pathology to Therapeutic Perspectives”

**DOI:** 10.3390/cells9112404

**Published:** 2020-11-03

**Authors:** Gerhild van Echten-Deckert

**Affiliations:** Life & Medical Science (LIMES) Institute for Membrane Biology and Lipid, Biochemistry at the Kekulé-Institute, University of Bonn, 53121 Bonn, Germany; g.echten.deckert@uni-bonn.de

It is an honor for us to dedicate this Special Issue to our dearest friend Lina Obeid, who was not only a pioneer in the field of sphingolipids, but also a remarkable personality. We lost her on November 19, 2019. There are no words to describe the deep pain we feel in losing Lina Obeid, an internationally recognized scientist, a wonderful mentor, ardent mother, and last but not least an empathic and cheerful friend. Among her numerous outstanding scientific achievements, I want to highlight her study published in 1993 in *Science*, where she showed for the first time that ceramide is a bioactive lipid that plays a role in apoptotic cell death [1]. This publication opened up a new era in sphingolipid research. Lina was also one of the first to recognize the key role of sphingosine kinase 1 (SK1) in cancer and to envision this enzyme as an anticancer target in p53-dependent tumors [2,3]. Last but not least, I want to mention the numerous excellent and helpful reviews she wrote, often together with her husband Yusuf Hannun, such as the one from 2018, which depicts and updates our knowledge on the critical roles of sphingolipid metabolism in physiology and disease [4]. Thank you, Lina.

Sphingolipids are ubiquitous components of cellular membranes [5]. Following their discovery in the brain and first description in 1884 by J.L.W. Thudichum [6], sphingolipids were largely overlooked for almost a century, perhaps due to their complexity and enigmatic nature [7,8]. It was with the discovery of sphingolipidoses, a series of inherited diseases caused by mutations of enzymes involved in sphingolipid degradation that sphingolipids returned to the limelight [9,10,11]. The essential breakthrough came decades later in the 1990s with the discovery that sphingolipids are not just structural elements of cellular membranes, but they are also intra- and extracellular signaling molecules [12]. It turned out that not only their complex carbohydrate head-groups, but especially their lipid backbones, including ceramide [1,13] and sphingosine-1-phosphate (S1P) [14] have selective physiological functions. As a result of this new concept, sphingolipids emerged as essential players in many pathologies including cancer [15,16], diabetes [17], neurodegenerative disorders [18], and autoimmune diseases [19]. The present issue reflects the evolvement of sphingolipids as bioactive signaling molecules that have unexpectedly eclectic functions in health, disease, and therapy. 

In cells, sphingolipids are generally lower in abundance than glycerolipids or cholesterol, representing less than 20% of total lipid mass [20]. The mammalian brain contains the largest amounts of sphingolipids in the body. Thus, it is not surprising that a rather large number of the contributions to this Special Issue refer to the role of sphingolipids in brain health and disease. 

The review by Lucaciu et al. highlights the role of S1P and its receptors in neurological disorders. This contribution provides a detailed picture of S1P metabolism and function in different cellular compartments. Most interestingly, the authors present an array of drugs targeting the S1P signaling pathway, which are being tested in clinical trials. 

Related to this review, the study of Alam et al. shows that S1P accumulation in neural cells exerts pathological effects that are receptor-independent. This group of researchers have previously shown that S1P is not always neuroprotective [21,22]. Interestingly, the harmful effects of S1P were also shown in beta-cells of the pancreas [23]. Of note, neurons and pancreatic beta-cells share important developmental transcriptional programs [24]. 

Rose-Mary Boustany’s research group studied the impact of sphingolipid metabolism in juvenile neuronal ceroid lipofuscinosis (now classified as CLN3 disease), which represents the most common form of neuronal ceroid lipofuscinoses (NCLs), a family of fatal, inherited pediatric neurodegenerative disorders [25]. The present contribution by Maloouf et al. proposes that flupirtine could be of use as a therapy for CLN3 disease and represents a first approach to define the mechanisms through which it may exert its actions. 

Two other contributions focus on sphingolipid metabolism and its role in the central nervous system. Zoicas et al. elucidate that in major depression and comorbid anxiety, the activity of enzymes that catalyze both ceramide generation and degradation, including sphingomyelinases and ceramidases, respectively, are elevated in particular brain regions. In a related context, Cosima Rhein’s research group has presented a novel mouse model of depression. In this model, overexpression of acid sphingomyelinase is restricted to the forebrain (see the contribution by Zoicas, Schumacher et al.).

However, the metabolism of ceramide is not only central to the brain, it has also been shown to play a major role in cellular and physiological processes including apoptosis, senescence, and inflammation [26]. The review by Duarte et al. focuses on ceramidases but also gives a detailed picture of ceramide metabolism and its involvement in numerous pathologies. Last, but not least, the authors discuss the usefulness of these enzymes as therapeutic tools. 

As mentioned above, diabetes and the metabolic syndrome are other human pathologies in which the role of sphingolipids is continuously being unraveled. Intriguingly, inhibition of ceramide synthesis in models of metabolic diseases prevents insulin resistance and diabetes and hence complications related to this disease [27]. In addition, C16-ceramide accumulation has been directly correlated with insulin resistance [28]. Guitton et al. present a detailed overview of the role of S1P in obesity and type 2 diabetes, whereas the contribution of Gurgul-Convey discusses in depth what is known so far about the involvement of sphingolipids in beta-cell physiology and pathophysiology, and their contribution to type 1 diabetes development and progression. 

Sphingolipids and cancer represent another important chapter with regard to the versatile functions of these molecules. In this volume, the review by Carrié et al. provides a very useful and important piece of information on the putative role of alterations of sphingolipid metabolism in tumorigenesis, progression and therapy of malignant melanoma. 

The key role of SK1 in cancer was a core area of Lina Obeid’s research [3]. Indeed, SK1 activity directly impacts the level of 3 bioactive sphingolipids; on the one hand it drives the formation of S1P while simultaneously clearing sphingosine, hence, impeding the generation of ceramide via the salvage pathway. It is therefore important to understand the mechanism of its regulation. The present Issue offers two contributions regarding this question. The contribution by Bonica et al. from Lina’s group provides a comprehensive overview of the transcriptional regulation of SK1, indicating the role of different transcriptions factors as well as of microRNAs in different systems in healthy and diseased states. Post-transcriptional and post-translational control of SK1 by G-protein coupled receptors (GPCRs) is one of the research topics examined by Meyer zu Heringdorf’s group. The latest findings of Blankenbach et al. on the Gq/11-mediated plasma membrane translocation and the activation of SK1 are part of this Special Issue. 

The study by Gräler’s group (Paul et al.) uncovers a new aspect of the role of S1P as an effective endothelial cell barrier stabilizing signaling molecule, which involves its secretion via the S1P-transporter Spinster homolog 2 (Spns2). 

Studies on the role of S1P and its receptors in myocardial function are presented by Wafa et al., where it appears that S1P acts as a double-edged sword. As shown and discussed by the authors, depending on the physiological conditions, S1P may exert a protective or a deleterious effect on cardiac function. 

Paola Signorelli’s research group has extensive experience regarding the role of sphingolipids in cystic fibrosis. Here, Mingione et al. provide evidence on how the controlled modulation of sphingolipid metabolism can function as a useful therapeutic strategy to overcome this inherited disease. 

The articles in this Special Issue are written by leading experts and cover many of the various roles played by sphingolipids in pathologies that are driven by perturbed sphingolipid metabolism. I am convinced that this fascinating lipid class will continue to be the subject of up-and-coming future discoveries, especially with regard to new therapeutic strategies. 
